# Impaired Prolactin-Lowering Effects of Metformin in Women with Polycystic Ovary Syndrome

**DOI:** 10.3390/jcm12175474

**Published:** 2023-08-23

**Authors:** Robert Krysiak, Karolina Kowalcze, Witold Szkróbka, Bogusław Okopień

**Affiliations:** 1 Department of Internal Medicine and Clinical Pharmacology, Medical University of Silesia, Medyków 18, 40-752 Katowice, Poland; wszkrobka@sum.edu.pl (W.S.); bokopien@sum.edu.pl (B.O.); 2 Department of Pediatrics in Bytom, Faculty of Health Sciences in Katowice, Medical University of Silesia, Stefana Batorego 15, 41-902 Bytom, Poland; kkowalcze@sum.edu.pl

**Keywords:** androgen excess, hyperprolactinemia, insulin resistance, metformin, pituitary, polycystic ovary syndrome

## Abstract

The effect of metformin on prolactin concentration seems to be sex-dependent. The aim of this study was to determine whether the androgen status modulates the impact of metformin on plasma prolactin levels in women. This study included two matched groups of prediabetic women with hyperprolactinemia: 25 with PCOS and 25 control subjects with androgen levels within the reference range and with normal ovarian morphology. Glucose homeostasis markers, prolactin, the remaining anterior pituitary hormones, sex hormones, SHBG and IGF-1 were determined before and after six months of metformin treatment. At baseline, both groups differed in LH, LH/FSH ratio, testosterone, FAI, DHEA-S, androstenedione and estradiol. Although metformin improved insulin sensitivity and increased SHBG in both study groups, these effects were more pronounced in control subjects than in women with PCOS. In control subjects, the drug decreased total and monomeric prolactin and increased LH. In women with PCOS, metformin reduced LH, LH/FSH ratio, testosterone and FAI. In the control group, the impact on total and monomeric prolactin positively correlated with their baseline levels and with the degree of improvement in insulin sensitivity, as well as negatively correlated with testosterone and FAI. In women with PCOS, treatment-induced changes in testosterone and FAI positively correlated with the changes in LH and LH/FSH ratio. The obtained results suggest that the prolactin-lowering properties of metformin are less pronounced in women with coexisting PCOS than in women with elevated prolactin levels, probably owing to the increased production of endogenous testosterone.

## 1. Introduction

Long-term prolactin excess is often complicated by obesity/overweight, insulin resistance and impaired glucose tolerance [1], disturbances commonly treated with metformin [2]. In numerous studies, metformin inhibited enhanced secretory function of human lactotropes [3,4,5,6,7]. The drug reduced prolactin levels, irrespective of the reason for prolactin excess, including in individuals with iatrogenic hyperprolactinemia [3,4,5,6], prolactin-secreting tumors [7], hyperprolactinemia secondary to traumatic brain injury [7] and prolactin excess related to empty sella syndrome [7], as well as in idiopathic hyperprolactinemia [7]. The drug was also found to potentiate a prolactin-lowering effect of small doses of bromocriptine, an agent commonly used to treat enhanced secretory function of lactotropes [8]. However, the effect of metformin was weak in subjects with dopamine agonist-resistant pituitary tumors [9] and absent in patients with an excess of macroprolactin (high-molecular-weight complexes of monomeric prolactin and immunoglobulins) [10]. The degree of reduction in prolactin levels depended on metformin dose and was most pronounced in individuals receiving high-dose treatment (2.55–3 g daily) [6]. The impact of metformin on prolactin concentration was also determined by sex and was statistically significant only in women [11]. Metabolic disturbances resulting from prolactin excess are effectively reversed by dopaminergic agents; however, owing to intolerance or contraindications, not all patients can be treated with these agents [12]. Some of these patients, particularly women with coexisting disturbances of glucose homeostasis, may be candidates for metformin treatment. Interestingly, metformin, even at high doses, does not seem to lead to prolactin deficiency. In normoprolactinemic subjects, the drug did not affect circulating levels of this hormone, while in hyperprolactinemic ones, its effect on lactotrope secretory function correlated with baseline prolactin levels [6,7]. In turn, inadequately high doses of dopamine agonists result in lactotrope hypofunction, the presence of which is associated with increased cardiovascular risk and impaired sexual functioning [13,14].

Polycystic ovary syndrome (PCOS) is the most common endocrinopathy in women of reproductive age. PCOS is also a leading cause of female hyperandrogenism and a state of relative estrogen excess [15,16]. The disorder often coexists with prolactin excess, although there are no convincing data that they are causally linked with each other [17]. Because of frequent disturbances in glucose homeostasis, women with PCOS often need to be treated with metformin [15,16]. To date, only two studies have investigated the effect of metformin on prolactin levels in women with PCOS, finding no changes in this hormone [18,19]. Unfortunately, the study populations included only women with PCOS, and some participants had the normoandrogenic phenotype of this disorder. Moreover, all included subjects were treated with only 1 g of metformin daily and were allowed to use oral contraceptives. However, indirect evidence supports the hypothesis that hormonal abnormalities characterizing PCOS may modulate metformin action on lactotrope function. Combined oral contraceptive pills [20] and estrogen replacement therapy [21] were found to potentiate the impact of metformin on plasma prolactin in hyperprolactinemic women without PCOS. In turn, metformin-induced reduction in prolactin levels in men was observed only if hyperprolactinemia was accompanied by low testosterone levels, and this effect inversely correlated with testosterone and calculated free testosterone levels [22].

Despite the frequent coexistence of PCOS and prolactin excess, as well as common use of metformin in women with PCOS, the question of whether metformin action on lactotrope secretory function is affected by coexisting PCOS has not been adequately addressed. Therefore, the aim of the present study was to compare the effect of high-dose metformin treatment on circulating prolactin levels in hyperprolactinemic women with classic PCOS and without this disorder, and to assess whether metformin action on lactotrope secretory function in women is modulated by androgen status. 

## 2. Materials and Methods

The study protocol was approved by the institutional review board and followed the principles of the Declaration of Helsinki. Written informed consent was obtained from each patient prior to enrollment, after the participants had been given information about the study and their rights. The results were reported in accordance with STROBE (Strengthening the Reporting of Observational Studies in Epidemiology).

### 2.1. Study Population 

This prospective matched cohort study enrolled 50 women, aged between 20 and 50 years, with prediabetes (fasting plasma glucose in the range between 100 and 126 mg/dL and/or plasma glucose 2 h after a glucose load of at least 140 mg/dL but below 200 mg/dL, despite complying with lifestyle modifications for at least 3 months), coexisting with mild to moderate hyperprolactinemia, defined as plasma prolactin levels in the range between 30 and 75 ng/mL. The study population consisted of two groups. The first group included 25 women with classic PCOS, diagnosed based on the presence of clinical and/or biochemical signs of hyperandrogenism and oligo-ovulation/anovulation after exclusion of other etiologies, such as congenital adrenal hyperplasia, androgen-secreting tumors, Cushing syndrome, use or abuse of androgenic/anabolic drugs, thyroid dysfunction and type 2 diabetes mellitus. Clinical hyperandrogenism was defined as the presence of hirsutism (modified Ferriman–Gallwey score equal to or greater than 8), persistent acne or androgenic alopecia. Hyperandrogenemia was diagnosed if free androgen index (FAI) exceeded 5%. Amenorrhea was defined as absence of menses for 3 months or longer, while oligomenorrhea was defined as a menstrual cycle longer than 35 days. In turn, the second group included 25 women with prediabetes not meeting any of the Rotterdam diagnostic criteria of PCOS (hyperandrogenism, oligo-anovulation and polycystic ovary morphology). This group of patients, serving as a control group, was selected from among 80 potential participants by a computer algorithm in order to obtain two study populations matched for age, body mass index (BMI), the homeostatic model assessment 1 of insulin resistance (HOMA1-IR) and prolactin levels. Because an a priori power calculation showed that a total of 23 patients in each group was required to obtain a power of 80% and a significance level of 5% if the hypothesis was a 20% between-group difference in total prolactin levels, our study was adequately powered. To minimize the effect of seasonal fluctuations in the outcome measures, similar numbers of patients were recruited in winter (*n* = 13), spring (*n* = 12), summer (*n* = 12) and autumn (*n* = 13).

Because severe prolactin excess and pituitary tumors require specific treatment, the presence of prolactinoma, pituitary tumor co-secreting prolactin and other hormones and pseudoprolactinoma excluded participants from the study. The remaining exclusion criteria were as follows: non-classic PCOS, macroprolactinemia, diabetes mellitus, thyroid or adrenal disorders, impaired renal or hepatic function, cardiovascular disease (except for non-pharmacologically treated grade 1 hypertension), malabsorption syndrome, any other serious disorder, pregnancy or lactation, any pharmacotherapy (except for antipsychotic drugs) and poor patient compliance.

### 2.2. Study Design

For the following six months, all participants received metformin. The dose of this agent was up-titrated from a starting dose of 850 mg once daily in week 1, to 850–1000 mg twice daily in weeks 2 and 3, and to a maximum dose of 850–1000 mg three times a day (2.55–3 g daily), introduced from week 4 onwards. To minimize the risk of adverse effects, metformin tablets were taken with, or just after, main meals. In addition to medical treatment, the participants were also encouraged to follow the therapeutic lifestyle changes. Treatment compliance was assessed by the pill counting method, while adherence to non-pharmacological recommendations was evaluated by analysis of individual dietary questionnaires.

### 2.3. Laboratory Assays

Venous blood samples were obtained from all patients on the first and last day of the study (in menstruating women, between days 2 and 5 of the cycle). They were collected from the antecubital vein at 8:00 a.m. after an overnight 12 h fasting and after the patient had been resting for a minimum of 30 min in the seated position. All experiments were conducted in duplicate by technicians blinded to the study protocol. Plasma glucose concentrations were determined using the Cobas Integra 400 plus (COBAS Integra 400 Plus, Roche Diagnostics, Basel, Switzerland). Plasma concentrations of insulin, prolactin, luteinizing hormone (LH), follicle-stimulating hormone (FSH), androgens (testosterone, dehydroepiandrosterone-sulfate (DHEA-S) and androstenedione), SHBG, estradiol and thyrotropin were measured by direct chemiluminescence using acridinium ester technology (ADVIA Centaur XP Immunoassay System, Siemens Healthcare Diagnostics, Munich, Germany). Prolactin was assayed both before (total prolactin) and after (monomeric prolactin) polyethylene glycol precipitation, carried out as previously described [23]. Macroprolactin content was calculated by subtracting monomeric prolactin from total prolactin. Plasma concentrations of adrenocorticotropic hormone (ACTH) and insulin-like growth factor-1 (IGF-1) were assessed by solid-phase enzyme-labelled chemiluminescent immunometric assays (Immulite, Siemens, Munich, Germany). HOMA1-IR was calculated as the product of fasting glucose levels (mg/dL) and insulin levels (mU/L), divided by 405. FAI was calculated by multiplying plasma testosterone in nmol/L by 100 and dividing by SHBG concentration in nmol/L.

### 2.4. Statistical Analysis

All data were natural log-transformed to correct for skews in the distribution. For quantitative data, between-group comparisons were performed using unpaired Student’s *t*-tests, while within-group comparisons were made using Student’s paired *t*-test. The χ^2^ test was used to compare qualitative variables. Bivariate relationships were analyzed using Pearson’s correlation coefficient. *p*-values, corrected for multiple comparisons, of less than 0.05 were considered significant. 

## 3. Results

At study entry, LH, LH/FSH ratio, testosterone, FAI, DHEA-S, androstenedione and estradiol were higher in women with PCOS than in control subjects. Both groups were comparable with respect to age, reasons for hyperprolactinemia, smoking, BMI, blood pressure, HOMA1-IR, and plasma levels of glucose, total prolactin, monomeric prolactin, macroprolactin, FSH, SHBG, thyrotropin, ACTH and IGF-1 (Table 1 and Table 2).

Metformin treatment was well tolerated, and no serious adverse effects were reported. Because all patients, who complied with treatment and dietary recommendations, completed the study, the results of all enrolled patients were statistically analyzed. Daily metformin doses in both groups were similar (2.78 ± 0.20 vs. 2.75 ± 0.21 g; *p* = 0.6074).

Metformin reduced glucose and HOMA1-IR and increased SHBG in both study groups. In control women, but not in women with PCOS, the drug decreased total and monomeric prolactin and increased LH. In the PCOS group, but not in control subjects, metformin reduced LH, LH/FSH ratio, testosterone and FAI. Levels of the remaining hormones remained unchanged over the entire study period. There were differences between follow-up values of HOMA1-IR, total prolactin, monomeric prolactin, testosterone, SHBG, FAI, DHEA-S, androstenedione and estradiol (Table 2). There were no differences between baseline and follow-up smoking habits, BMI and blood pressure. Both groups differed in the percentage changes from baseline in HOMA1-IR, prolactin (total and monomeric), LH, LH/FSH ratio, testosterone, SHBG and FAI (Table 3).

In control subjects, the impact on prolactin positively correlated with baseline prolactin levels (total prolactin: r = 0.51 (*p* < 0.0001); monomeric prolactin: r = 0.55 (*p* < 0.0001)) and treatment-induced changes in HOMA1-IR (total prolactin: r = 0.38 (*p* = 0.0068); monomeric prolactin: r = 0.42 (*p* = 0.0006)), as well as inversely correlated with baseline testosterone (total prolactin: r = −0.32 (*p* = 0.0352); monomeric prolactin: r = −0.38 (*p* = 0.0088)) and baseline FAI (total prolactin: r = −0.35 (*p* = 0.0105); monomeric prolactin: r = −0.40 (*p* = 0.0008)). In women with PCOS, the impact of metformin on testosterone and FAI positively correlated with the changes in LH (testosterone: r = 0.40 (*p* = 0.0004); FAI: r = 0.46 (*p* = 0.0002)) and with the changes in the LH/FSH ratio (testosterone: r = 0.38 (*p* = 0.0015); FAI: r = 0.41 (*p* = 0.0006)). The remaining correlations were insignificant.

## 4. Discussion

The obtained results allow us to draw three general conclusions. Firstly, the inhibitory effects of metformin on various types of overactive pituitary cells were independent of one another. The drug reduced prolactin levels only in the control women, while its impact on LH levels (and LH/FSH ratio) differed between the two groups. Moreover, in women with PCOS, there were no correlations between the impact of metformin on secretory function of lactotropes and gonadotropes. Secondly, in line with previous observations [6,7,24,25], metformin decreased anterior pituitary hormones only if their baseline secretion was increased. In neither treatment group did metformin reduce thyrotropin, ACTH or IGF-1, the baseline levels of which were within the reference range. Lastly, follow-up values of the assessed hormones did not drop below the lower limits of normal, despite high-dose metformin treatment. This observation, as well as previous findings that only 2.55–3 g of metformin daily inhibits secretory function of the pituitary gland [6,24], are arguments in favor of the relatively low sensitivity of anterior pituitary cells to metformin and suggest that metformin treatment is not associated with a risk of pituitary hypofunction.

The major finding of the present study was that PCOS attenuated the impact of metformin on plasma prolactin. In contrast to the women having this disorder, in the control group, the drug reduced elevated prolactin levels, although the average follow-up values of this hormone were still above the upper limit of normal. The decrease in both total and monomeric prolactin and no changes in macroprolactin indicate that the drug reduced production of the monomeric hormone but had no effect on the production and/or metabolism of complexes of prolactin and immunoglobulins (mainly immunoglobulin G) [26]. The different effects of metformin in the two treatment arms may be, at least partially, explained by differences in the androgen status of patients. The impact of treatment on the plasma levels of both total and monomeric prolactin in women without PCOS inversely correlated with testosterone and FAI, which is used to estimate physiologically active free testosterone in women [27]. This observation is in line with the results of previous studies including male subjects. In individuals with early-onset male-pattern hair loss, considered the equivalent of PCOS in men [28], metformin produced a neutral effect on plasma prolactin [29], while in hypogonadal men with prolactin excess, the decrease in plasma prolactin was observed only in patients not receiving exogenous testosterone [22]. The current study is the first to show similar relationships between hyperandrogenism and the absence of prolactin-lowering properties in women. It should be underlined that testosterone and FAI in PCOS are usually only mildly or moderately increased, as well as that these levels are much lower than in men [30]. Thus, it seems that the impact of metformin on lactotrope function is modulated by relatively small changes in testosterone production. Beyond testosterone, the second factor correlating with the impact of metformin on plasma prolactin was the degree of reduction in HOMA1-IR, an established marker of insulin resistance/sensitivity [31], which cannot be attributed to baseline differences in prolactin excess, body mass and glucose homeostasis (both groups were matched for prolactin levels, BMI and HOMA1-IR).

Owing to the study protocol, the mechanisms explaining the differences in metformin action on prolactin secretion remain speculative. The most probable explanations for the inhibitory effect of PCOS on the prolactin-lowering properties of metformin are, however, interactions at the level of the pituitary adenosine 5′-monophosphate-activated protein kinase (AMPK) pathway or the tuberoinfundibular dopaminergic system. In line with the first explanation, androgen receptors are present in the cytosol and nuclei of lactotropes [32]. Pituitary cells, including lactotropes, are characterized by the presence of the catalytic subunit of AMPK [33]. Many biological effects of metformin are mediated by activation of the AMPK pathway, including its action on pituitary cells [33,34,35]. Moreover, in women with PCOS, endogenous testosterone was found to inhibit the AMPK pathway [36]. Interestingly, AMPK is an important sensor of energy status and maintains cellular energy homeostasis, and its decreased activity is associated with impaired insulin sensitivity [34,35]. Thus, the opposite effect of metformin and elevated testosterone levels may explain a weaker impact of metformin on HOMA1-IR in women with PCOS as compared to women without PCOS.

There are, however, arguments in favor of an alternative explanation. The pituitary is the brain region with the greatest differences in the distribution of D_2_/D_3_ receptor binding between males and females [37]. Metformin may modulate the endogenous dopaminergic tone in obese women with PCOS, simultaneously providing an amelioration of the insulin resistance status [38]. Moreover, the activity of tuberoinfundibular dopaminergic neurons was inhibited by testosterone [39]. Finally, metformin action on thyrotropin secretion in women with subclinical hypothyroidism and PCOS depended on the degree of activation of tuberoinfundibular dopaminergic neurons [40].

Despite a stronger effect on plasma prolactin, the follow-up levels of this hormone in women without PCOS were still elevated, which suggests that the impact of metformin on lactotrope function in this population is moderate. This effect was less pronounced than that induced by treatment with dopamine agonists (bromocriptine, quinagolide and cabergoline), the drugs of choice in patients with prolactin excess [12]. Unfortunately, there are no head-to-head studies comparing the prolactin-lowering properties of monotherapy with a dopamine agonist and metformin. However, unlike metformin recipients in the current study, bromocriptine [41,42] and cabergoline [43] monotherapy led to a significant decrease in prolactin in hyperprolactinemic women with concomitant PCOS. Furthermore, cabergoline administered to women with this syndrome together with low-dose metformin was superior to low-dose metformin alone in reducing prolactin levels [18,19]. These findings indirectly indicate that also in women with PCOS, dopaminergic agents are more potent in reducing monomeric hyperprolactinemia than metformin. Interestingly, although bromocriptine reduced serum prolactin concentration in rodents with experimentally induced PCOS, circulating levels of this hormone were still markedly higher than in control animals [44]. This finding may suggest that dopaminergic agents are less effective in decreasing prolactin production in women with PCOS than in other groups of hyperprolactinemic patients, and that baseline tuberoinfundibular dopaminergic transmission may partially determine the impact of different drugs on lactotrope secretory function. It seems that this interesting and clinically relevant question requires further research.

Another interesting observation of our study was the opposite effect of metformin on LH levels. The decrease in women with PCOS, accompanied by a decrease in the LH/FSH ratio, which is in line with previous observations of other research teams [45,46], is probably a consequence of the inhibitory effect on overactive gonadotropes. It was found that the inhibitory effect of metformin on gonadotropin secretion was mediated by pituitary AMPK, and gonadotropes were pituitary cells with abundant expression of this enzyme [33]. The presence of correlations between the impact of metformin on LH and the LH/FSH ratio and on testosterone and FAI indicates that the decrease in LH secretion in this group of women contributed to a reduction in testosterone production. In turn, the increase of LH in the control group is probably secondary to the decrease in prolactin levels. This explanation is supported by the presence of correlations between the impact on prolactin and on LH in the control subjects but not in women with PCOS. Through inhibition of hypothalamic gonadotropin-releasing hormone pulsatile secretion, prolactin excess reduces gonadotropin secretion and may have an additional direct inhibitory effect on ovarian function [47]. Because the decrease in prolactin and the subsequent increase in LH in subjects without PCOS were only moderate, an indirect effect on ovarian function may be counterbalanced by a direct inhibitory effect of this agent on ovarian steroidogenesis [48].

Although women with and without PCOS differed in estradiol levels, contrary to testosterone, they remained at a similar level throughout the study and did not correlate with the pituitary effects of metformin. This observation was somewhat surprising in light of our previous studies, reporting a stimulatory effect of estrogen replacement therapy in postmenopausal women [21] and of ethinyl estradiol-containing oral contraceptives in women in reproductive age [20] on lactotrope secretory function in metformin-treated patients. Moreover, this finding contradicts observations that metformin inhibits aromatase activity in peripheral tissues [49,50]. Because the modulatory effects of estrogen were observed in women with low baseline estrogen levels and in women with supraphysiological concentrations of these hormones, but not in females with mildly increased estrogen production, it is possible that the association between estrogen and the prolactin-lowering effect of metformin may be determined by the estrogen status of patients. Alternatively, our findings may be explained by the fact that circulating estrogen levels poorly reflect their local production in various tissues [51].

The current study has several limitations that should be considered when interpreting our findings. Owing to a small number of participants, the results should be interpreted as hypothesis-generating rather than definitive conclusions. The study included only women with classic PCOS (phenotypes A and B), and therefore, it is uncertain whether phenotypes C and D are also associated with an impaired effect of metformin on prolactin production. Concentrations of monomeric prolactin and macroprolactin were determined using a less accurate precipitation method, while the reference technique is gel filtration chromatography [26]. Lastly, because the study does not provide insight into the cellular and molecular aspects of interactions between metformin and sex hormones, mechanisms underlying the obtained results still remain unclear and require further research.

## 5. Conclusions

Although metformin improved insulin sensitivity in both study groups, this effect was less pronounced in women with coexisting classic PCOS. In patients with normal androgen levels and normal ovarian morphology, the drug reduced total and monomeric prolactin, while a decrease in LH, LH/FSH ratio, testosterone and FAI was observed only in subjects with PCOS. The impact on total and monomeric prolactin in women with normal androgen levels depended on the degree of improvement in insulin sensitivity and on baseline testosterone and FAI. Our observations suggest that the presence of PCOS may attenuate the impact of metformin on lactotrope secretory function via increased testosterone production. Because of the pilot nature of this research, the results of the current study should be confirmed in longitudinal observational studies with adequate sample sizes.

## Figures and Tables

**Table 1:** Baseline characteristics of patients.

Variable	PCOS Group	Control Group	*p*-Value
Number (*n*)	25	25	-
Age (years)	35 ± 8	36 ± 7	0.6402
Reasons for hyperprolactinemia (iatrogenic/traumatic brain injury/empty sella syndrome/idiopathic) (%)	52/20/20/8	48/24/16/12	0.7846
Smokers (%)/Number of cigarettes a day (*n*)/Duration of smoking (months)	40/11 ± 5/140 ± 42	40/10 ± 6/146 ± 39	0.8834
BMI (kg/m^2^)	24.2 ± 4.8	23.8 ± 5.1	0.7443
Systolic blood pressure (mmHg)	129 ± 18	126 ± 16	0.4635
Systolic blood pressure (mmHg)	85 ± 6	84 ± 5	0.5251

Unless otherwise stated, the data are presented as the mean ± standard deviation. Abbreviations: BMI—body mass index; PCOS—polycystic ovary syndrome.

**Table 2:** The impact of metformin on glucose homeostasis markers and plasma hormone levels in hyperprolactinemic women with and without polycystic ovary syndrome.

Variable	PCOS Group	Control Group	*p*-Value (PCOS vs. Controls)
Glucose (mg/dL)			
*Baseline*	110 ± 9	109 ± 10	0.7118
*Follow-up*	100 ± 10	97 ± 10	0.2942
*p-value (follow-up* vs. *baseline)*	**0.0005**	**0.0001**	-
HOMA1-IR			
*Baseline*	3.9 ± 1.2	3.7 ± 1.1	0.5419
*Follow-up*	3.0 ± 0.8	2.1 ± 0.6	**<0.0001**
*p-value (follow-up* vs. *baseline)*	**0.0031**	**<0.0001**	-
Total prolactin (ng/mL)			
*Baseline*	55.0 ± 10.4	53.9 ± 11.2	0.7205
*Follow-up*	51.0 ± 12.0	43.0 ± 8.9	**0.0101**
*p-value (follow-up* vs. *baseline)*	0.2139	**0.0004**	-
Monomeric prolactin (ng/mL)			
*Baseline*	51.6 ± 10.0	50.3 ± 10.8	0.6608
*Follow-up*	47.8 ± 10.6	39.6 ± 8.5	**0.0041**
*p-value (follow-up* vs. *baseline)*	0.1985	**0.0003**	-
Macroprolactin (ng/mL)			
*Baseline*	3.4 ± 1.5	3.6 ± 1.9	0.6814
*Follow-up*	3.2 ± 2.0	3.4 ± 1.6	0.6980
*p-value (follow-up* vs. *baseline)*	0.6908	0.6890	-
LH (U/L)			
*Baseline*	5.0 ± 2.1	2.8 ± 1.6	**0.0001**
*Follow-up*	3.5 ± 1.7	4.1 ± 1.8	0.2316
*p-value (follow-up* vs. *baseline)*	**0.0078**	**0.0096**	-
FSH (U/L)			
*Baseline*	3.4 ± 1.6	3.2 ± 1.5	0.6505
*Follow-up*	3.8 ± 2.0	4.0 ± 2.1	0.7316
*p-value (follow-up* vs. *baseline)*	0.4387	0.1277	-
LH/FSH ratio			
*Baseline*	1.5 ± 0.6	0.9 ± 0.4	**0.0001**
*Follow-up*	0.9 ± 0.4	1.0 ± 0.3	0.3221
*p-value (follow-up* vs. *baseline)*	**0.0001**	0.3221	-
Testosterone (nmol/L)			
*Baseline*	2.9 ± 0.7	1.1 ± 0.4	**<0.0001**
*Follow-up*	2.5 ± 0.6	1.2 ± 0.3	**<0.0001**
*p-value (follow-up* vs. *baseline)*	**0.0350**	0.3221	-
SHBG (nmol/L)			
*Baseline*	35.0 ± 11.2	37.5 ± 10.4	0.4175
*Follow-up*	41.8 ± 12.0	48.7 ± 11.6	**0.0441**
*p-value (follow-up* vs. *baseline)*	**0.0437**	**0.0008**	-
FAI (%)			
*Baseline*	8.3 ± 1.6	2.9 ± 1.0	**<0.0001**
*Follow-up*	6.0 ± 1.8	2.5 ± 1.1	**<0.0001**
*p-value (follow-up* vs. *baseline)*	**<0.0001**	0.1848	
DHEA-S (μmol/L)			
*Baseline*	10.8 ± 4.0	6.5 ± 2.2	**<0.0001**
*Follow-up*	9.0 ± 3.7	5.6 ± 1.6	**0.0001**
*p-value (follow-up* vs. *baseline)*	0.1051	0.1046	**-**
Androstenedione (nmol/L)			
*Baseline*	8.0 ± 2.0	4.0 ± 1.5	**<0.0001**
*Follow-up*	7.5 ± 2.0	3.8 ± 1.3	**<0.0001**
*p-value (follow-up* vs. *baseline)*	0.3812	0.6167	**-**
Estradiol (pmol/L)			
*Baseline*	260 ± 90	170 ± 75	**0.0004**
*Follow-up*	282 ± 101	204 ± 85	**0.0048**
*p-value (follow-up* vs. *baseline)*	0.4202	0.1402	-
Thyrotropin (mU/L)			
*Baseline*	2.8 ± 1.0	2.8 ± 0.9	1.0000
*Follow-up*	2.6 ± 0.9	2.5 ± 1.0	0.7118
*p-value (follow-up* vs. *baseline)*	0.4609	0.2704	-
ACTH (pg/mL)			
*Baseline*	40 ± 15	38 ± 16	0.6505
*Follow-up*	37 ± 12	36 ± 14	0.7874
*p-value (follow-up* vs. *baseline)*	0.4387	0.6402	-
IGF-1 (ng/mL)			
*Baseline*	202 ± 56	192 ± 62	0.5523
*Follow-up*	212 ± 60	214 ± 53	0.9011
*p-value (follow-up* vs. *baseline)*	0.5453	0.1838	-

Follow-up and baseline results were compared in the same group. Statistically significant results are marked in bold. Reference values for young women in the early follicular phase: glucose: 70–99 mg/dL; HOMA1-IR: <2.0; total prolactin: 5.0–29.0 ng/mL; monomeric prolactin: 4.0–26.0 ng/mL; macroprolactin: 2.0–4.0 ng/mL; LH: 2.2–8.5 U/L; FSH: 3.1–10.0 U/L; testosterone: 0.4–2.0 nmol/L; SHBG: 30–140 nmol/L; FAI: <5%; DHEA-S: 2.0–11.0 μmol/L; androstenedione: 1.2–8.0 nmol/L; estradiol: 180–650 pmol/L; thyrotropin: 0.4–4.5 mU/L; ACTH: 15–70 pg/mL; IGF-1: 85–300 ng/mL. Abbreviations: ACTH—adrenocorticotropic hormone; DHEA-S—dehydroepiandrosterone-sulfate; FAI—free androgen index; FSH—follicle-stimulating hormone; HOMA1-IR—homeostatic model assessment 1 of insulin resistance ratio; LH—luteinizing hormone; PCOS—polycystic ovary syndrome; SHBG—sex hormone-binding globulin.

**Table 3:** Percentage changes from baseline in the investigated variables in the study population.

Variable	PCOS Group	Control Group	*p*-Value
Δ Glucose	−9 ± 5	−11 ± 6	0.2066
Δ HOMA1-IR	−23 ± 11	−43 ± 20	**0.0001**
Δ Total prolactin	−7 ± 5	−20 ± 11	**<0.0001**
Δ Monomeric prolactin	−7 ± 4	−21 ± 10	**<0.0001**
Δ Macroprolactin	−6 ± 5	−6 ± 7	1.0000
Δ LH	−30 ± 16	46 ± 20	**<0.0001**
Δ FSH	12 ± 19	25 ± 28	0.0067
Δ LH/FSH ratio	−40 ± 20	11 ± 8	**<0.0001**
Δ Testosterone	−14 ± 10	9 ± 11	**<0.0001**
Δ SHBG	19 ± 12	30 ± 16	**0.0084**
Δ FAI	−28 ± 14	−14 ± 10	**0.0002**
Δ DHEA-S	−17 ± 12	−14 ± 12	0.3812
Δ Androstenedione	−7 ± 8	−5 ± 5	0.2945
Δ Estradiol	8 ± 16	19 ± 25	0.0700
Δ Thyrotropin	−7 ± 8	−11 ± 10	0.1249
Δ ACTH	−8 ± 11	−5 ± 10	0.3180
Δ IGF−1	5 ± 14	11 ± 18	0.1946

The data are presented as the mean ± standard deviation. Statistically significant results are marked in bold. Abbreviations: ACTH—adrenocorticotropic hormone; DHEA-S—dehydroepiandrosterone-sulfate; FAI—free androgen index; FSH—follicle-stimulating hormone; HOMA1-IR—homeostatic model assessment 1 of insulin resistance ratio; IGF-1—insulin-like growth factor-1; LH—luteinizing hormone; PCOS—polycystic ovary syndrome; SHBG—sex hormone-binding globulin.

## Data Availability

The data that support the findings of this study are available from the corresponding author upon reasonable request.

## Abbreviations

ACTH—adrenocorticotropic hormone; AMPK—adenosine 5′-monophosphate-activated protein kinase; BMI—body mass index; DHEA-S—dehydroepiandrosterone-sulfate; FAI—free androgen index; FSH—follicle-stimulating hormone; HOMA1-IR—homeostatic model assessment 1 of insulin resistance ratio; IGF-1—insulin-like growth factor-1; LH—luteinizing hormone; PCOS—polycystic ovary syndrome; SHBG—sex hormone-binding globulin.
